# Investigating the effects of social information on spite in an online game

**DOI:** 10.1017/ehs.2024.18

**Published:** 2024-04-12

**Authors:** Robin Watson, Thomas J. H. Morgan, Rachel L. Kendal, Julie Van de Vyver, Jeremy Kendal

**Affiliations:** 1 Durham Cultural Evolution Research Centre, Anthropology Department, Durham DH1 3LE, UK; 2 Department of Anthropology, Durham University, Dawson Building, South Road, Durham DH1 3LE, UK; 3 School of Human Evolution and Social Change, Arizona State University, 900 South Cady Mall, Tempe, AZ 85287, USA; 4 Institute of Human Origins, Arizona State University, 777 E University Drive, Tempe, AZ 85287, USA; 5 Psychology Department, Durham University, Upper Mountjoy, South Road, Durham DH1 3LE, UK; 6 Durham Research Methods Centre, Faculty of Social Sciences & Health Arthur Holmes Building, Durham University, Durham DH1 3LE, UK

**Keywords:** spite, social learning, social behaviour, punishment, altruism

## Abstract

While humans are highly cooperative, they can also behave spitefully. Yet spite remains understudied. Spite can be normatively driven and while previous experiments have found some evidence that cooperation and punishment may spread via social learning, no experiments have considered the social transmission of spiteful behaviour. Here we present an online experiment where, following an opportunity to earn wealth, we asked participants to choose an action towards an anonymous partner across a full spectrum of social behaviour, from spite to altruism. In accordance with cultural evolutionary theory, participants were presented with social information that varied in source and content. Across six conditions, we informed participants that either the majority or the highest earner had chosen to behave spitefully, neutrally or altruistically. We found an overall tendency towards altruism, but at lower levels among those exposed to spite compared with altruism. We found no difference between social information that came from the majority or the highest earner. Exploratory analysis revealed that participants’ earnings negatively correlated with altruistic behaviour. Our results contrast with previous literature that report high rates of spite in experimental samples and a greater propensity for individuals to copy successful individuals over the majority.

## Graphical abstract

**Figure:**
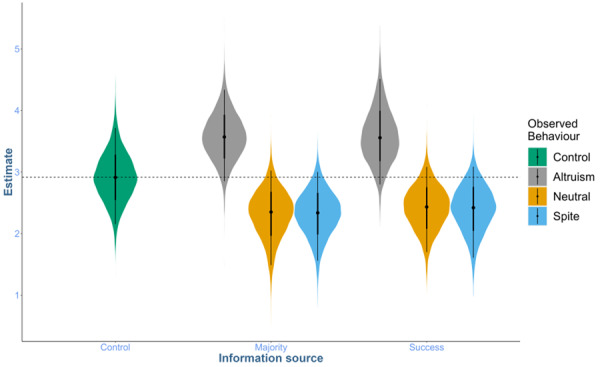

**Social media summary:** Social transmission of spite and altruism: altruism is reduced following exposure to spiteful social information

## 1. Introduction

Compared with other animals, humans are unusually cooperative (Fehr & Rockenbach, 2004; Henrich & Muthukrishna, 2021). It is well established that altruism, incurring a net cost to the actor's lifetime fitness (West et al., 2007; West & Gardner, 2010), can evolve through inclusive fitness effects (Hamilton, 1964) or be sustained through reciprocity (Trivers, 1971) or reputational effects (Nowak & Sigmund, 2005). Spiteful actions, resulting in a net cost to both individuals in an interaction (West et al., 2007) are rare in animals but surprisingly common in humans. Theory has distinguished between two different kinds of spite: evolutionary spite and functional or psychological spite (Jensen, 2010).

Evolutionary spite describes cases where spiteful actions are directed towards non-relatives, which benefit one's relatives. Evolutionary spite can evolve through inclusive fitness if the actor is less related to the recipient than the average relatedness in the population (Hamilton, 1964; Wilson, 1975). Examples of evolutionary spite are extraordinarily rare. This is partly because it is difficult to conclusively demonstrate that seemingly spiteful behaviour could not provide direct fitness benefits at a future point (Foster et al., 2001; Patel et al., 2020), but also because there are probably few scenarios where harming others represents the best strategy to assist your relatives (West & Gardner, 2010; but see Gardner et al., 2007). We do not consider evolutionary spite further.

Instead, we focus on functional or psychological spite (henceforth ‘spite’). Such behaviour is mutually costly in the short term and may or may not indirectly increase the actor's fitness in the long term (Jensen, 2010). For instance, engaging in spite may improve your relative payoff if the cost to other individuals is greater than the cost to yourself (Jensen, 2010). Spite is like punishment (both involve inflicting harm on others), but is distinguished by an individual's motivation. Punishment is used to affect the future behaviour of the target (Balliet et al., 2011; Boyd et al., 2003) such that the harm caused is a means to an end. For spite, the harm caused is the end in itself – any resulting benefits are secondary (Jensen, 2010). As an illustration of the difference between spite and punishment, consider two experiments which found that chimpanzees were most likely to remove their partner's access to food in response to theft (Jensen et al., 2007), but capuchins were equally likely to remove access to a partner's food if the partner had more, irrespective of how it was obtained (Leimgruber et al., 2016). Here, chimpanzees appeared to engage in punishment whereas capuchins appeared to engage in spite.

Forms of spite may evolve through indirect reciprocity by deterring others’ aggression (Johnstone & Bshary, 2004), or by an anticorrelation effect where spiteful individuals are inclined to interact with non-spiteful individuals in small groups (Bruner & Smead, 2022; Smead & Forber, 2012). Spite may also be a response to intense local competition (Gardner & West, 2004) or have evolved as a by-product of costly punishment (Hauser et al., 2009). In human participants, spite (directed at high earners) was more common when there were larger imbalances between individuals’ earnings (Dawes et al., 2007; Prediger et al., 2014; Raihani & McAuliffe, 2012). Such spite is more common when the inequality could have arisen from luck or cheating, rather than being earned (D. Fehr, 2018; Gee et al., 2017).

Within humans, anecdotes of mutually costly behaviours are common. For example, Mui (1995) describes several anecdotes of successful farmers or business owners having their possessions destroyed and Scott (1992) notes that ‘[spite] is a familiar aspect of divorce negotiations’ (p. 646). Online trolling and abuse are another common form of spite (e.g. Synnott et al., 2017), although the payoffs, motivations and costs associated with such actions may be complex and difficult to identify. Thus, a popular approach is to examine spite though behavioural experiments, where participants are offered the option to harm another player, usually by reducing their earnings.

In one such experiment, the authors identified a ‘substantial incidence of nasty behaviour … where spiteful acts could be covered by random destruction’ (Abbink & Sadrieh, 2009, p. 6) which the same authors then supported in a later experiment (Abbink & Herrmann, 2011). Later experiments (but see, Blackwell & Diamond, 2017) investigating factors such as resource scarcity (Prediger et al., 2014), the presence of eyes (Baillon et al., 2013) and the choice set presented to participants (L. Zhang & Ortmann, 2016) concluded their findings to be consistent with Abbink and Herrmann (2011). In answering the question ‘are people willing to pay to burn other people's money?’, Zizzo and Oswald (2001, p. 52) concluded the short answer to be ‘yes’. These claims, however, may be exaggerated on account of several experimental design features that we discuss below in addition to the file-drawer effect, whereupon null results are less likely to be published than significant results (Rosenthal, 1979).

First, contrary to standard definitions of spite (West et al., 2007), in many experiments actors are permitted to inflict a cost to a recipient without incurring a cost to themselves (as in Abbink & Sadrieh, 2009; Blackwell & Diamond, 2017; Zhang & Ortmann, 2016). Second, participants’ choices are limited to behaving spitefully or doing nothing (as in Abbink & Herrmann, 2011; Abbink & Sadrieh, 2009; Baillon et al., 2013; Blackwell & Diamond, 2017; D. Fehr, 2018; Prediger et al., 2014; Zizzo & Oswald, 2001), or they are presented with separate opportunities to practice only spite or only altruism (L. Zhang & Ortmann, 2016). This may conflict with some participants’ preferences to compensate, rather than punish, other participants (FeldmanHall et al., 2014) or spite may be selected because it is novel and more appealing (in the experimental setting) than doing nothing. More generally, it remains unclear why individuals may choose to be spiteful with no clear incentive. One possibility that we explore in this experiment is that spite may spread via social information.

Cultural evolutionary theory suggests that it is adaptive for humans to make selective use of social information in the form of social learning strategies (Kendal et al., 2018; Laland, 2004; Morgan et al., 2012). For example, in an unfamiliar environment or when the adaptive value of a new behaviour is unclear, selection may favour learners that use indirect cues of adaptive behaviour (sometimes called ‘context’ biases), for example by copying the majority or a successful or prestigious individual, (Henrich & Gil-White, 2001; Jiménez & Mesoudi, 2019; McElreath & Henrich, 2003; Sarin & Dukas, 2009). While generally adaptive, these strategies leave room for the spread of maladaptive or costly behaviours such as spite, as learners acquire practices without directly assessing their adaptive value. Further, certain kinds of social information, such as that rich in social or emotional content, may also be more likely to be remembered and transmitted, a phenomenon described as ‘content bias’ (Kendal & Watson, 2023).

There is experimental evidence that altruism and punishment can be copied. Participants have been shown to increase their altruism in social dilemma games in response to observing altruism displayed by a high-status individual (Gächter & Renner, 2018; Kumru & Vesterlund, 2010) or by altruistic individuals from another group (Romano & Balliet, 2017). Cross-culturally, there is evidence that altruism can be influenced by context-specific social norms (Henrich et al., 2010). However, when also shown the payoffs of others, individuals appear to engage in payoff-biased copying and reduce their altruism (Burton-Chellew et al., 2017b; Burton-chellew & Amico, 2021; Molleman et al., 2014; Watson et al., 2021). In ultimatum games, a theoretical model showed that a form of payoff-biased social learning resulted in average offerings of between 40 and 50% (Zhang, 2013).

There is also experimental evidence that punishment is copied. Individuals were more likely to engage in punishment after learning that other participants favoured punishment (FeldmanHall et al., 2018) or that punishment and cooperation were the normative behaviour (Li et al., 2021). The prevalence of antisocial punishment (punishment directed at altruistic individuals) also varied between cultures (Bruhin et al., 2020; Herrmann et al., 2008). In competitive football, players were more likely to engage in intentional fouling or aggressive play if they associated with peers or coaches who endorsed it (Kabiri et al., 2020; Malete et al., 2013). Other forms of antisocial or aggressive behaviours (which may reflect, or be motivated by, spite) have been shown to be predicted by association with other victims or perpetrators. These include using cheating tools in online games (Kim & Tsvetkova, 2022), the use of excessive force by police officers (Ouellet et al., 2019) and violent crimes (Tracy et al., 2016). Nonetheless, to our knowledge, few (if any) experiments have directly assessed the spread of spite via social learning.

Here, to examine the social transmission of spite, we consider the effects of social information content and source on participants’ social behaviour. Regarding information content, experiments have found evidence that social and emotional content are particularly transmissible (Mesoudi et al., 2006; Stubbersfield et al., 2017), while analysis of sensationalist newspaper headlines across a 300 year period found that stories frequently concerned altruism and cheater detection (Davis & McLeod, 2003).

Regarding the information source, we consider conformity (or copy-the-majority; Boyd & Richerson, 1985; Morgan & Laland, 2012) and copy-the-successful (McElreath & Henrich, 2003; Sarin & Dukas, 2009) social learning strategies. Both have been documented in a variety of contexts (reviewed in Kendal et al., 2018; Kendal & Watson, 2023), including studies investigating altruism (Burton-Chellew et al., 2017a; Burton-Chellew & Amico, 2021; Watson et al., 2021). Note, however, that some studies have found no effect of information source on transmission. For example, the likeability of quotes was not influenced by whether the quote was attributed to a famous or less famous author (Acerbi & Tehrani, 2018) and participants’ later recall of narratives depended more strongly on the content of the narrative than whether the story was told by a speaker with a (previously rated) highly prestigious accent (Berl et al., 2021).

### 1.1. Research questions

In our study, we expand upon the methodology of previous experiments to assess spite's prevalence when it is (1) costly to the participant and (2) offered as a choice alongside altruism. Under these experimental conditions, we test whether social information – varying in source and content – affects participants’ subsequent behaviour. To our knowledge, no previous studies have investigated the social transmission of spite. In doing so, we contribute to previous studies that explore the proximate explanations for costly spite. We address the following research questions (RQ):

**RQ1: To what extent is spiteful behaviour exhibited in our experiment?** Based on the lowest and highest rates of spite observed in previous experiments, we predict that between 10 and 40% of participants will behave spitefully. However, we note that such experiments rarely consider costly spite and/or offer participants the choice to be altruistic and so in our experiment rates may be lower.

**RQ2: Does social information enabling the use of conformity or copy-the-successful strategies affect social behaviour?** As there is stronger evidence for the effect of success-biased social influence than conformity on cooperative behaviour, we predict that copy-the-successful information will exert a stronger influence than conformity information on participant's behaviour (whether spiteful or altruistic).

The RQs were established before completing the experimental design and data collection. After looking at the data, we decided to conduct an unplanned, exploratory analysis to determine whether social behaviour was influenced by personal earnings accrued in an earlier part of the experiment.

## 2. Methods

### 2.1. Design

The experiment consisted of two parts. In the first part, participants played a game in which they earned points. In the second part, participants were either given social information (Table 1) or assigned to an asocial control group that received no social information, before having the opportunity to donate (altruism) or withdraw (spite) points from an anonymous partner at a cost to themselves. We ran six social information conditions in a between-participants 3 × 2 factor design (Table 1). Factor 1 was the source of social information (the majority of previous participants or the most successful previous participant), while Factor 2 specified the behaviour of the source towards their partner (spite, altruism or neutral). The experiment received ethical approval from the Anthropology ethics committee at Durham University. All data, code, and supplementary material can be found at https://osf.io/ekmuj/.

**Table 1.** Conditions and sample sizes. Social information presented to participants varied by the information source (Factor 1) and the source's behaviour towards the partner player (Factor 2). All social information was fictitious but presented to be perceived as real by the participants.

		Source behaviour (Factor 2)			
Social information		Reduced points of partner (*spite*)	Did not change points of partner (*neutral*)	Increased points of partner (*altruism*)	Asocial control
Information source (Factor 1)	Most successful	47	57	41	54
	Majority	53	43	51	

### 2.2. Materials and procedure

The experiment was conducted online using the experimental platform Dallinger (2022) and participants were recruited on Amazon's Mechanical Turk (MTurk). Once participants joined the experiment, a screen indicated that they were awaiting a second participant. After a short delay, the experiment began. Throughout, participants were deceived into thinking that a second participant was simultaneously taking part in the experiment. To enhance believability, randomised time delays were used throughout the experiment to suggest that they had to wait for the other participant to catch up.

In part one (see Supporting Information, SI 1), participants played a five-round game with a bot (they were aware they were playing with a bot). The purpose of this was for participants to accumulate points to be used in part two. It was important for participants to feel that they had earned their points to alleviate concerns of ‘house-money’ effects, where participants are more reckless with points or money that they do not feel is theirs (Abbink & Sadrieh, 2009, but see Harrison, 2007). Participants were told that the points they had obtained by the end of the experiment would be converted to a bonus payment but not how much each point was worth.

In each round of part one, participants were given 10 points and could send any amount of this to the bot. The bot then sent between 0 and 12 points to the player, equal to the value that the participant had sent plus a randomly generated number between −2 and 5. This wide range was used to prevent participants from easily working out the pattern. The participant's score for the round was determined by the points that they received from the bot plus the points they kept for themselves.

In part two (see SI 2), participants were told that either they or the other participant would be assigned randomly to the ‘decider’ role and could pay points to increase or decrease the other participant's score. In reality, the other participant was a bot, and so the human participants were always assigned to the ‘decider’ role. It was made clear to the participant that their decision was one-shot, and the recipient would have no opportunity to respond.

It cost the participant one point for every three points donated or withdrawn from their partner's score, up to a maximum of 10 points’ cost for a 30-point change to the partner's score. The participant indicated their choice using a slider, which updated to show how their choice would affect their own and their partner's scores. This 3:1 ratio of partner's score-change to cost was chosen based on previous studies employing costly punishment (Fischbacher & Fehr, 2004; Rand & Nowak, 2011). Changing the partner's score represented a monetary cost for participants, as their points at the end of the experiment were converted into a bonus payment.

In each of the social conditions and before making their decision, participants received experimentally manipulated information about one or more previous participants’ score-change decisions. Depending on the source behaviour condition (Factor 2), the participant received information stating that previous participants: ‘did not change their partner's score’ (neutral); ‘increased their partner's score’ (altruism); or ‘decreased their partner's score’ (spite). The information source condition (Factor 1) was stated to be either ‘the majority of previous participants’ (conformity) or ‘the highest scoring participant in previous games’ (copy-the-successful).

Following participant's one-shot score change decision, we collected free-text responses to gain insight into their reasoning about the experiment (see SI 3). As a comprehension check, participants were asked to specify whether they had chosen to increase, decrease or not change their partner's earnings. Participants were debriefed, and the deception employed in the experiment explained (see SI 4). They were reminded of their right to withdraw at this point (5 did). Finally, demographic information was collected, and participants were asked to rate their level of understanding of the game on a Likert scale from 1 (did not understand at all) to 10 (perfectly understood).

### 2.3. Participants

Data collection took place online via MTurk between the 22 and 28 of July 2021. Participants were recruited in blocks of 75 and were randomly assigned to a condition. Participants who did not complete the experiment or who requested their data be removed were excluded, leaving 346 participants. Because conditions were assigned randomly, there was some imbalance between conditions (Table 1). Owing to a software error, two participants had two responses associated with their ID. In these cases, the first response (as determined by time created) was kept and the other observation was discarded.

Of those who provided demographic information, the median age was 32 years (interquartile range, IQR = 9) with 197 identifying as male, 75 as female and two as non-binary. A total of 253 participants identified as White, 28 as Asian, 34 as Black African or Caribbean, 12 as Latin American and six as mixed race; three withheld this information. All participants earned a minimum of $0.35 for completing the experiment with a further $0.60 earnable as a bonus. Participants earned $0.65 on average and the experiment took around 5 minutes to complete.

### 2.4. Data analysis

Analyses were conducted in R studio version 4.1.0 (R Core Team, 2021). We used Bayesian linear models to analyse the data, implemented in the *rethinking* package (McElreath, 2020). Bayesian methods combine prior beliefs with data to produce ‘posterior distributions’ – mathematical descriptions of our knowledge about parameters or hypotheses. Here, posterior distributions for parameter values (for example, the *β* values for predictors) were estimated using Markov Chain Monte Carlo (MCMC) methods. In MCMC methods, multiple chains of values are created that converge on likely parameter values and, at equilibrium, produce values according to their posterior probability (i.e. their plausibility given the data and prior probabilities). As such, independent values drawn from chains at equilibrium are mathematically equivalent to values drawn from the posterior distribution for each parameter. A large number of these values, often called ‘samples’, can then be plotted or summarised to learn about the parameter being estimated. For instance, the median sample can be used as a point estimate, while the proportion of samples that fall within a given region is equal to the probability that the true value is within that region. The samples can also be used to generate predictions, including uncertainty, regarding outcomes in hypothetical situations. In this work we used four chains to generate at least 3000 independent samples for each parameter.

The 95% prediction interval (PI) is the range of the samples, excluding the highest and lowest 2.5%. It defines the most central region, which has a 95% chance of containing the true value, thus it is sometimes referred to as a ‘central credible interval’. Where a parameter's 95% PI excludes zero, we consider this to be strong evidence of that parameter having an effect.

To further assess the evidence for different effects, we compare models with and without parameters according to their WAIC (widely applicable information criteria) value, which provides an estimate of each model's out of sample predictive ability. Such model comparison can provide evidence that certain variables are predictive of the outcome, rather than overfitted to the data. Lower WAIC values indicate better out-of-sample predictions.

While Bayesian models allow prior information to be included in the form of priors, we adopt a common approach of using weakly regularising priors which makes the model sceptical of extreme estimates, but otherwise minimally influences its conclusions. For further discussions on Bayesian modelling and MCMC methods see McElreath (2020) and Kruschke (2015).

We termed the outcome variable ‘social behaviour’. A value of 10 indicated that the participant had increased their partner's score by the maximum amount (i.e. paying 10 points to increase their partners score by 30) and −10 that they had decreased their partner's score by the maximum amount (i.e. paying 10 points to decrease their partner's score by 30).

To address RQ1, we used an *intercept-only model* to generate a posterior distribution for social behaviour across all conditions:

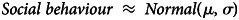


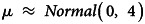


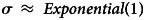

where *Social behaviour* is modelled with a normal distribution, with mean *μ* and standard deviation *σ*. To address RQ2, we used the following *condition model*:

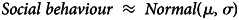


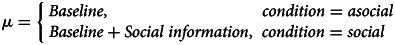


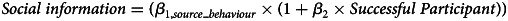


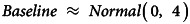


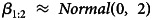


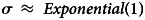

Here, *successful participant* has a value of 1 in the social conditions where the source is a successful participant, but 0 where the source is the majority. Thus, the effect of the social information (altruistic, neutral or spiteful) is estimated by 
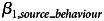
 when the source is the majority, but it is multiplied by (1+*β*_2_) when the information source was the most successful previous participant. As such, *β*_2_ reflects the influence of a successful individual relative to the majority.

Our model structure was motivated by our experimental design. We did not include an independent main effect of information source because our focus is only on the modulating effect of an information source on the source behaviour and information source and content were not separable in our experiment. However, we can still compare the relative effects of the two information sources via our *β*_2_ parameter.

Finally, we conducted an unplanned, exploratory analysis to evaluate the extent to which each participant's score in part one affected their part two behaviour. For this, we modified the *condition model* by allowing baseline to be a function of score (i.e. *μ = Baseline* + *Social information* +  *β*_3_ × *Score*). The score variable was standardised and *β*_3_ was assigned a prior of *Normal*(0, 2).

## 3. Results

### 3.1. To what extent is spiteful behaviour exhibited in our experiment? (RQ1)

**Figure 1.**
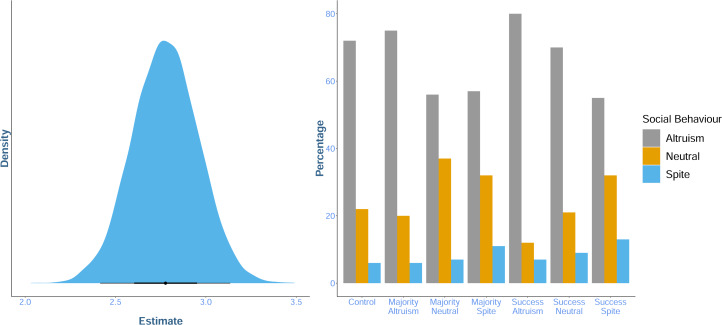
Left: Density plot of values from the posterior distribution of the mean behaviour in the intercept model. The point indicates the mean of the distribution and lines indicate the 68 and 95% prediction intervals (PIs). Positive numbers indicate altruistic behaviour. Right: Descriptives from the experiment data. Percentages of participants within each experimental condition (e.g. ‘Majority Altruism’ was the majority who displayed altruistic behaviour) opting for altruistic (grey), neutral (yellow) and spiteful (blue) behaviour.

**Very little**: the estimates from the posterior distribution of the *intercept model* were positive, indicating that participants chose to be mostly altruistic (Figure 1 left; mean: 2.77; 95% PI, 2.42, 3.13; SD: 3.38; 95% PI, 3.14, 3.63). In addition (Figure 1 right), the descriptive frequency of altruism (66.47%) was far higher than that of neutral (25.14%) or spiteful behaviour (8.38%). The low rates of spite were inconsistent with our predictions.

### 3.2. Does social information enabling the use of conformity or copy-the-successful strategies affect social behaviour? (RQ2)

**Figure 2.**
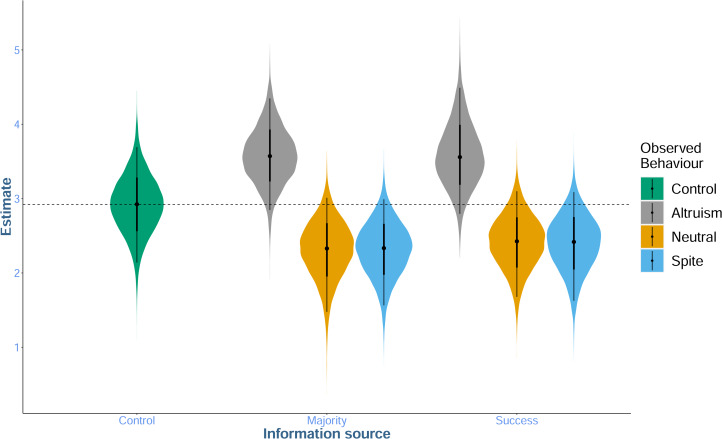
Ten thousand predictions of mean social behaviour across experimental conditions drawn from the posterior distribution of the condition model. Points show the mean of the sampled distribution, and the surrounding lines display the 68 and 95% prediction intervals (PIs). Colours indicate the social behaviour participants saw: altruistic (grey), neutral (yellow) or spiteful (blue) and the *x*-axis shows the source of the information (the majority of or the most successful prior participant). The dashed line indicates the control condition mean (3), displayed for comparison.

**Yes, modestly**: compared with the control condition, we found some evidence that social information indicating that previous participants had behaved altruistically increased participant's altruistic behaviour. However, we found only weak evidence that information indicating spiteful or neutral behaviour had an effect, and it did so by decreasing altruistic behaviour (Table 2). There was no evidence of a difference in the effect of information content between information sources (i.e. whether the social information came from the majority of other participants, or the single most successful participant; Figure 2.). The *condition* model was moderately favoured by WAIC compared with the *intercept* model (WAIC: *intercept* = 1827.6, SE = 27.06, weight = 0.2; *condition* = 1824.8, SE = 28.78, weight = 0.8), indicating that including the condition predictor slightly improved the model's out of sample predictions. Predicted social behaviour from the *condition* model is shown in Figure 2.

**Table 2.** Mean, 95% prediction interval (PI) and overall percentage of the posterior distribution that has the same sign (positive or negative) as the mean for the parameters associated with altruistic, spiteful and neutral social information. This provides evidence for a difference between social conditions and the asocial control condition.

Condition	Mean (95% PI)	Percentage of posterior in direction of the mean
Altruism	0.89 (2.6, −0.81)	87.28
Spite	−0.36 (1.34, −2.2)	65.45
Neutral	−0.35 (1.35, −2.23)	64.55

To estimate differences between the different social conditions, contrasts were generated between the posterior distribution of the parameter associated with altruistic social information (*β*_1,*altruisim*)_ and the posterior distribution of the parameter associated with spiteful behaviour (*β*_1,*spite*_). This provided strong evidence that altruistic social information increased participant's altruism relative to spiteful social information (mean = 1.25, 95% PI = [0.3, 2.35], percentage of samples in direction of mean = 99.62%). Thus, while evidence for a difference between the social conditions and asocial baseline varied from moderate to weak, there was strong evidence for a difference between the effects of altruistic and spiteful social information.

### 3.3. Exploratory analysis of the influence of participant's earnings on social behaviour

**Figure 3.**
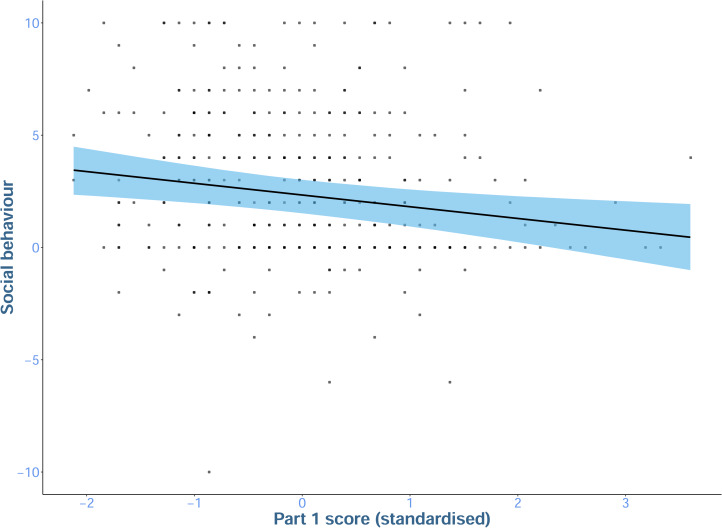
Mean social behaviour predicted by a participant's score in part one (high values on the *y*-axis indicate more altruistic behaviour). The line shows the mean of the predictions, and the shaded region represents the 95% prediction interval (PI). Points show raw data. Predictions are drawn from the majority neutral condition. Note that the part one score is the participant's score prior to making their score change decision as the decider.

Predictions from the *score* model (which included both conditions and participant's part 1 scores, Figure 3) indicated that participants who earned more in part one tended to be less altruistic in part two than those who earned less in part one. Model comparison supported the inclusion of participant's part one score into the model. The model that included part one score accounted for 95% of the WAIC weight between the *score*, *intercept* and *condition* models (Table 3) indicating that adding score to the model improved its predictions out of sample, although the effect of part one score was small.

**Table 3.** WAIC (widely applicable information criteria) values and model weights for the three models fit to the data. The standard error difference provides the standard error of the difference between each model and the model with the lowest WAIC score while the standard error indicates the standard error of the associated WAIC score. Note that the score model also included effects of the different conditions.

Model	WAIC	SE	Standard error difference	Weight
*Score*	1818.2	30.12	0	0.95
*Condition*	1824.6	28.74	6.4	0.04
*Intercept*	1827.7	27.09	9.6	0.01

### 3.4. Participant's understanding of the experiment

Overall, participants self-reported ratings indicated a generally good understanding of how the experiment worked (rated from 1 to 10: median = 8, IQR = 3), which suggested that participants did not feel confused during the experiment. However, participants were also asked to report whether and how they had changed their participant's score in part two of the study. Of the 222 participants that provided a response, among altruistic participants, 98/135 (72%) correctly reported that they had increased their partner's score; among neutral participants, 61/71 (85.91%) correctly reported that they had done nothing; and among spiteful participants, 9/16 (56.25%) correctly reported that they had decreased their partners’ score. The lower comprehension among spiteful participants could indicate that they were confused about how the decider role worked, or that they were reluctant to self-report that they had been spiteful. While we cannot rule out one possibility over the other, we note that median self-reported understanding ratings were largely equal between those that were altruistic, spiteful and made no change to their partner's score (altruistic: median = 8, IQR = 4; neutral: median = 9, IQR = 2; spiteful: median = 8, IQR = 3.25). In addition, among all spiteful participants, most (13) opted not to provide a response to the comprehension question, which may indicate a reluctance to self-report their decision. However, to confirm that our primary conclusions were not biased by poor comprehension, we repeated our main analyses on data containing only participants that answered the comprehension question correctly. We opted to also retain those who provided no answer, as this was a substantial number of participants (118). These results (presented in SI 5) did not qualitatively differ from our main findings.

## 4. Discussion

Our experiment investigated the prevalence of spiteful behaviour (RQ1) and the influence of social information (RQ2) on participant's social behaviour. Overall, we found extremely low rates of spite but reduced altruistic behaviour following exposure to social information indicating that prior participants were spiteful, relative to a case where the social information indicated that prior participants were altruistic. An exploratory analysis found that the degree of altruism in part two of the experiment was negatively related to participant's points earned in part one.

The strong inclination for altruism over spite (RQ1) runs counter to several experiments showing evidence for spite in humans (Abbink & Sadrieh, 2009; Baillon et al., 2013; Prediger et al., 2014; Zizzo & Fleming, 2011). Our results were closest to the rates of spite (around 10%) reported by Abbink and Herrmann (2011) in their ‘open’ condition, where spiteful decisions could not be hidden by the random loss of points. The low rates of spite in our experiment were surprising, as participants were fully anonymous. The degree of altruism in our study was similar to dictator games where offerings average around 28% (Engel, 2011). However, our experiment differs in an important way. In a dictator game, dictators allocate a percentage of a sum of points to a partner (Engel, 2011), where they gain what they choose to keep, whereas in our experiment deciders paid points to benefit/cost the receiver three times as much. Consequently, the selfish option is different between our experiment (do nothing) and dictator games (keep entire sum of points).

With respect to the effect of social information (RQ2), we found moderate reductions in altruistic behaviour after being exposed to spiteful or neutral behaviour compared with altruistic social information. This difference might suggest that social learning can promote the spread of spiteful behaviour. However, it is important to recall that our model predicts that most social behaviour, even when participants viewed spiteful or neutral information, was altruistic. Thus, our results support the more tentative conclusion that, setting aside payoff effects on fitness, spiteful social information may reduce the generosity of altruistic acts, but not that such information would necessarily strongly increase the frequency of spiteful behaviour.

Participants were not affected by whether the social information source was the population majority or the most successful individual. This is consistent with experimental work suggesting stronger influences of information content than the source on the transmission of narratives (Berl et al., 2021). Consistent with other studies, the overall effect of social information on behaviour was small (Street et al., 2018; reviewed in Morin et al., 2021). If the social information content was sufficiently memorable by itself, perhaps the source was unimportant. Indeed, the adaptive value of model-based social learning strategies is predicted to be low when the payoff consequences of behaviour can be assessed (McElreath & Henrich, 2003), as was the case in our experiment.

Our exploratory analysis found that the degree of altruism displayed in part two of the experiment was negatively related to participant's score (wealth) from part one of the experiment. Economic game experiments have found mixed results regarding cooperation and wealth. Some find a negative relationship (Erkal et al., 2011), some no relationship (Hofmeyr et al., 2007) and others find that wealthy participants contribute less in relative terms but equally in absolute terms than less wealthy participants (Buckley & Croson, 2006). With respect to spite, although Zizzo and Oswald (2001) found no relationship between being wealthy and being spiteful, other experiments have shown that spite was directed at wealthier players (Dawes et al., 2007) or that punishment was a response more to unfavourable inequity than to experiencing a loss (Raihani & McAuliffe, 2012). In contrast to our results, previous work has found that spiteful money burning was most common when resources were scarce than when they were abundant, although this may have reflected an influence of competition (Prediger et al., 2014). Further work could explore the impact of wealth on spiteful behaviour more explicitly.

Unlike many experimental studies (for example, Abbink & Herrmann, 2011; Baillon et al., 2013; Prediger et al., 2014), we offered participants the full range of actions along a spectrum from highly spiteful to highly altruistic, where the same degrees of altruism and spite were equally costly to enact. Offering only spite may inflate its prevalence in experiments if spite is enacted for its novelty or if participants that would have otherwise chosen to be altruistic are restricted from doing so by the experimental design. Consistent with this, Feldman-Hall et al. (2014) found after receiving an unfair offer many participants preferred to increase their own score rather than punish the unfair offer.

Our design ensured that spiteful behaviour was costly to the actor (Abbink & Sadrieh, 2009; Blackwell & Diamond, 2017; Kimbrough & Reiss, 2012; L. Zhang & Ortmann, 2016). Although non-costly harmful behaviour is still interesting, it is not as challenging to explain as costly spite. Furthermore, the actor's knowledge that they are absolved of negative repercussions does not reflect many real-world scenarios where there is a transparent cost to the act.

There are some caveats to the study worth noting. While participant's self-rated understanding of the experiment was high across all experimental conditions, only 56.25% accurately reported acting spitefully, while altruism and neutral behaviour were reported much more accurately (altruism, 72%; neutral, 85.91%). This may reflect participant confusion (Ferraro & Vossler, 2010) or a desire to hide their spiteful behaviour for social desirability concerns. The precise reason for the mismatch between observed and reported spite is unclear from the data collected, but rates of intentionally spiteful behaviour may be even lower than 8%. However, the main conclusions drawn in Sections 3.1–3.3 did not change when we repeated our analysis with participants that answered the comprehension question incorrectly excluded (SI 5). We also note that our experimental design may have inadvertently promoted altruism through framing (Gerlach & Jaeger, 2016). Part one resembled a trust game (Johnson & Mislin, 2011) and we referred to the other participant as their ‘partner’ throughout, which may have primed participants to behave altruistically. The wording we used to describe the successful participant (‘the highest scoring participant in previous games’) referred to the part one score but was also a little ambiguous, which may have weakened its effect on participants’ behaviour.

It is important to be cautious in generalising from a sample of MTurk participants. Although a review by Rand (2012) indicated that economic game results from MTurk samples are typically comparable with those conducted in person, cross-cultural work has previously identified that economic game behaviour (Henrich et al., 2010) and antisocial punishment (Bruhin et al., 2020) vary according to demographic factors such as market integration. Cooperative behaviours can also vary within cultures (Lamba & Mace, 2011). It is therefore highly likely that spite may also vary across cultures. Our one-shot, anonymous study design may be limited to simulating online interaction contexts such as social media or online gaming or those occurring in anonymous contexts such as voting or high population-density settings. Finally, we acknowledge that our use of deception is potentially problematic. We deceived participants by recruiting only one real participant and providing fictitious social information. We did this to avoid recruiting two participants but only using the data from one (the decider). While there is evidence that deception does not appear to influence participants’ responses in experiments (Rahwan et al., 2022), deceiving participants risks eroding trust in experimental instructions (Charness et al., 2022). We suggest that our use of deception is unlikely to have biased our results (as we included delays to simulate a real two-player interaction) but agree that deception should not be widely used (Charness et al., 2022) and will avoid deception in any future studies.

Future work may focus on other mechanisms by which spite may culturally evolve such as competition (Gardner & West, 2004) or through desires to improve one's relative payoffs (Jensen, 2010). Indeed, experiments including a competitive component (mock auction: Kimbrough & Reiss, 2012) or competitive cues (resource scarcity: Prediger et al., 2014) found greater levels of spite than we observed in our study. However, few experimental studies have explicitly investigated the role of competition on spiteful behaviour by way of experimental comparison (but see Barker & Barclay, 2016). In accordance with functional spite, which includes cases of tangible long term indirect benefits (Jensen, 2010), it would be interesting to compare scenarios where spite offers no chance of future benefits (as in our experiment) with those where indirect future benefits are possible. A direct comparison between conditions where participants are restricted to spiteful behaviour or nothing vs. those where they are also offered altruism may be useful to test our suspicion that this may have influenced previous experimental results. Investigating real-life spiteful behaviour, perhaps making use of existing large datasets, may also facilitate greater understanding of the proximate factors that explain when people are spiteful.

In summary, the results of our experiment support two main conclusions. Firstly, when participants are offered the choice between altruism and (costly) spite in an anonymous one-shot game, spiteful behaviour is rare. This is consistent with evolutionary theory suggesting that spiteful behaviour is probably rare in nature. Second, exposure to spiteful or neutral compared with altruistic social information reduced altruism. This may be particularly relevant for real-world scenarios where there is exposure directed towards extreme models, for example by social media algorithms. Further, there was no evidence of an influence of the information source on social behaviour when comparing information about the majority behaviour with information about the behaviour of the most successful individual.

## Research transparency and reproducibility

The data associated with this research are available at https://osf.io/ekmuj/.
